# The Effects of Short-Term Synaptic Depression at Thalamocortical Synapses on Orientation Tuning in Cat V1

**DOI:** 10.1371/journal.pone.0106046

**Published:** 2014-08-26

**Authors:** Aylin Cimenser, Kenneth D. Miller

**Affiliations:** 1 Harvard Medical School, Boston, Massachusetts, United States of America; 2 Department of Physics, Boston University, Boston, Massachusetts, United States of America; 3 Center for Theoretical Neuroscience, College of Physicians and Surgeons, Columbia University, New York, New York, United States of America; 4 Department of Neuroscience, College of Physicians and Surgeons, Columbia University, New York, New York, United States of America; 5 Swartz Program in Theoretical Neuroscience, College of Physicians and Surgeons, Columbia University, New York, New York, United States of America; University of Southern California, United States of America

## Abstract

We examine the effects of short-term synaptic depression on the orientation tuning of the LGN input to simple cells in cat primary visual cortex (V1). The total LGN input has an untuned component as well as a tuned component, both of which grow with stimulus contrast. The untuned component is not visible in the firing rate responses of the simple cells. The suppression of the contribution of the untuned input component to firing rate responses is key to establishing orientation selectivity and its invariance with stimulus contrast. It has been argued that synaptic depression of LGN inputs could contribute to the selective suppression of the untuned component and thus contribute to the tuning observed in simple cells. We examine this using a model fit to the depression observed at thalamocortical synapses *in-vivo*, and compare this to an earlier model fit based on *in-vitro* observations. We examine the tuning of both the conductance and the firing rate induced in simple cells by the net LGN input. We find that depression causes minimal suppression of the untuned component. The primary effect of depression is to cause the contrast response curve to saturate at lower contrasts without differentially affecting the tuned vs. untuned components. This effect is slightly weaker for *in-vivo* vs. *in-vitro* parameters. Thus, synaptic depression of LGN inputs does not appreciably contribute to the orientation tuning of V1 simple cells.

## Introduction

Much of the stimulus selectivity of simple cells in layer 4 of cat primary visual cortex (V1), including their tuning for stimulus orientation, can be understood from the feedforward input they receive along with cellular and synaptic nonlinearities [1]–[27]. Factors that may play a role in determining orientation tuning include the pattern of inputs the cells receive from the lateral geniculate nucleus (LGN) of the thalamus [5]; feedforward inhibition driven by LGN inputs [20], [24], [26]; suppression of voltage variability with increasing stimulus contrast, which changes the cell’s input/output function [17], [19]; and synaptic depression [27], [28]. Intracortical excitation affects the gain of simple-cell responses but does not seem to alter the orientation tuning induced by these other factors [6], [7] (see also in rodents: [29], [30]).

The arrangement of LGN inputs alone cannot account for orientation tuning. The net input driven by a simple cell’s LGN cells can be decomposed into an orientation-untuned component and a tuned component, which for a drifting grating stimulus correspond respectively to the mean and the temporal modulation of the input [20], [24]. Both components increase with contrast, so that the peak LGN input (mean plus modulation) in response to a high-contrast stimulus at the null orientation (orthogonal to the preferred) should be higher than that for a low-contrast preferred stimulus [20], [26]. Yet most V1 cells respond little to a high-contrast null stimulus while responding robustly to a low-contrast preferred stimulus [17], [31]–[33].

Two factors appear to suppress null relative to preferred responses. First, voltage responses to a null stimulus are weaker, relative to preferred, than predicted from the arrangement of LGN inputs. A null stimulus evokes depolarization that grows with contrast, but even at the highest contrast this depolarization is rarely if ever larger than the preferred-orientation voltage response at low (4%) contrast (Fig. 5A of [17]). Recurrent amplification of preferred responses may contribute to this. Considering LGN input alone, at any given contrast the mean LGN-induced input should be equal for the null and the preferred orientations [20]. Experimentally, the LGN-input-induced mean voltage response to the null is estimated to be only about 70% of that to the preferred (Fig. S3B of [19]). Second, the firing rate to a null stimulus is strongly suppressed relative to this voltage response, typically remaining slightly suppressed relative to spontaneous firing across all stimulus contrasts, so that the ratio of preferred to null firing rate strongly increases with stimulus contrast [17], [31]. The suppression of null spiking response is largely explained by an observed reduction in voltage variability with increasing contrast, which causes the mean null voltage response to remain a constant number of standard deviations of the voltage noise below spike threshold, keeping spiking probability (the firing rate) constant across contrasts [17], [19].

It has been argued that synaptic depression at geniculocortical synapses could suppress null voltage response and, along with the arrangement of LGN inputs, explain orientation tuning [28]. Here we reexamine this issue, using a model fit to the weaker synaptic depression seen *in-vivo* [34] as well as one fit to the stronger depression seen *in-vitro* [35].

## Materials and Methods

We modeled the spatial arrangement of the relay cells in LGN that connect to a V1 simple cell as in previous studies [20]. We used measured LGN firing rates and included synaptic depression, either fit by us to *in-vivo* experimental studies [34] as described below, or using a previous fit [23] to an *in-vitro* experimental study [35]. We modeled the tuning of the total LGN input – the total LGN-evoked excitatory conductance – to a V1 simple cell under three conditions: 1. No depression at thalamocortical synapses, 2. Depression, using *in-vivo* fit parameters 3. Depression, using *in-vitro* fit parameters. We also modeled the tuning of simple-cell firing rates that would be elicited by these conductances. We focused only on tuning, ignoring response amplitude (that is, we present tuning curves normalized to the response at the preferred orientation and maximal contrast studied), because tuning but not amplitude seems to be determined simply by feedforward input and its processing at the synapse and the postsynaptic cell, independently of intracortical excitation [6], [7], [29], [30] (excepting possible effects of intracortical excitation on voltage variability, which influences spike rate tuning, but this effect is incorporated in our spiking model as discussed further below).

### Determining the LGN firing rate

To determine orientation tuning curves at varying contrasts, we used LGN firing rates from experimental data (Dataset S1) that is kindly provided to us by Chong Weng and Jose-Manuel Alonso (SUNY). These data consist of the responses of seven LGN X-cells to drifting sinusoidal gratings in anesthetized cat LGN. The temporal frequency of the gratings was 1.6 Hz and the spatial frequency of the grating was.28 cycles/degree. The sinusoidal gratings were presented at contrasts: 3%, 6%, 12%, 24%, 48%, 72% and 96%.

We took the firing rate for a given LGN cell in response to a grating of a given contrast to be given by the cell’s cycle-averaged peri-stimulus time histogram (PSTH; the average firing rate as a function of time across one cycle of the drifting grating) for gratings of that contrast, averaged over 300 stimulus cycles. The phase of the PSTH for a grating of a given orientation was matched to the phase of the grating as it passed over the cell’s receptive field center (locations of LGN receptive field centers are described below). We also compared the performance of a linear rectified model fit to the PSTH, constructed as follows. Rectified sinusoids of the form 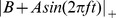 were constructed, where 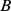 was the background firing rate, taken to be the mean firing rate at 3% contrast; 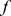 was the temporal frequency of the stimulus; 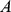 was the amplitude; and 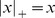, 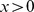; 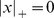 otherwise. The amplitude, 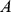, was adjusted so that the rectified sinusoid and the cycle-averaged PSTH had the same Fourier amplitude at the stimulus frequency.

The LGN firing rates used in fitting the depression model are described below, in the Materials and Methods subsection “Fitting the synaptic depression model”.

### Constructing LGN spike trains

For a given time-dependent firing rate of an LGN cell, spike timings were constructed from an inhomogeneous Poisson process with an absolute refractory period of 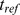 = 1 msec (or a homogeneous Poisson process in the case of background firing, when firing rates are not changing in time). The problem of generating spike trains with a specified refractory period was first studied by [36] and further analysis was done by [37]–[41]. For an absolute refractory period, the correction is simple: if the firing rate at time 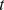 is 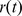, then the average fraction of trials that exhibit refractoriness at 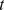 is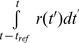. Thus, if the firing rate when not refractory – the “free firing rate” – is 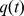, then the observed firing rate is equal to the free firing rate times the percentage of time not refractory:
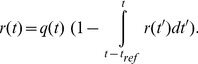
(1)We used a discretized version of this formula, given 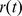 from the data, to compute 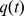; computed the next spike time after the last refractory period from an inhomogeneous Poisson process with rate 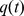; and set spike probability to zero for 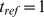 msec after each spike. Note that when the firing rate is a constant, the free firing rate can be written as:
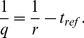
(2)For fitting the model to the data of Boudreau and Ferster [34], we used bins of 0.1 msec duration. For the remaining analysis, we estimated the firing rates using bins of 1 msec duration.

### Spatial Organization of LGN

The LGN was constructed with four overlying sheets [42] as in [20]. Each sheet covered the same 6.8°×6.8° area of the visual field. A sheet consisted of two lattices with 30×30 ON cells and 30×30 OFF cells. The two lattices were offset by one-half of the lattice spacing. A cell’s receptive field center location corresponded to its lattice position.

### Connections of relay cells in LGN to a simple cell in V1

A simple cell receptive field was modeled as a Gabor function [5], [43]: a sinusoidal oscillation of 0.8 cycles/degree multiplied by Gaussian envelopes parallel to and perpendicular to the direction of oscillation. We used the “broadly tuned” receptive field parameters of [20]: letting *x* and *y* be the directions parallel and perpendicular to the oscillation, respectively, the Gaussians in the Gabor had standard deviations 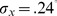 and 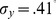. This results in a receptive field with 1.85 subfields (defined as the ratio of the length of the Gaussian parallel to the oscillation to one half-cycle of the oscillation, where the length of a Gaussian is defined as the distance between the points where it takes 5% of its peak value). The orientation of the receptive field was aligned with the LGN grid. The Gabor function was chosen to be symmetric about the center of the receptive field.

We determined the thalamocortical connection strengths to the model simple cell from this Gabor function receptive field stochastically as in [20]. This gives a time-invariant strength 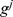 for the connection from the 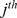 LGN neuron to the cortical cell. In addition, each strength is modulated by a time-varying factor 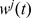 determined by synaptic depression, as explained below. The time dependence of the AMPA conductances for the 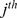 synapse were modeled as differences of simple exponentials for each presynaptic spike, weighted by the value of the depression factor 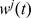 at the time of the spike:
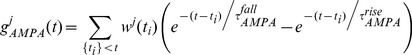
(3)with corresponding time constants 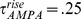 msec, 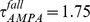 msec [20], where 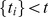 represents the presynaptic spike timings, 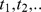, up to time 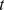. The time course of NMDA conductances was also modeled with exponentials, except that the decay of NMDA conductance was modeled as a double exponential with a fast and a slow time constant:



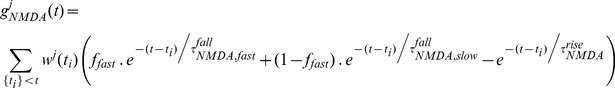
(4)The values were chosen from [22] as 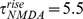 msec, 
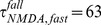
 msec, 
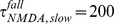
 msec and 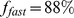. Voltage dependence of the NMDA conductance was ignored. The total conductance evoked by the 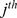 synapse was: 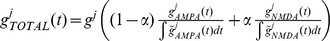 where 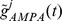 is 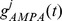 evaluated with a single presynaptic spike and 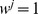, and similarly for 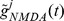. 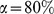 is the ratio of NMDA to AMPA conductances.

### Distribution of transmission delays from LGN to cortex

Boudreau and Ferster [34] classified cortical cells with response latencies less than 2.3 msec as monosynaptic cells, and provided a distribution of these response latencies. Reasoning that the difference in response latencies reflects differences in transmission delays between LGN and cortex, we randomly picked the transmission delay between a given LGN neuron and the model cortical neuron from the distribution mentioned above. This is accomplished by shifting the contribution to the PSP from an individual LGN neuron by the corresponding delay, and ensures that the response latency of the model cortical neuron is equal to the average latency of the monosynaptic cells studied in Boudreau and Ferster.

### Modeling synaptic depression

Depression is modeled as a factor 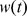 for each synapse that varies between 0 and 1, reflecting the degree of depression.

*In-vivo*: Depression was modeled with a calcium-dependent recovery time as suggested in previous studies [44]. The idea behind this model is that as calcium accumulates, the time constant for recovery becomes smaller, thereby speeding up the process of recovery from depression. In mathematical terms, between two spikes, the change in Calcium concentration, 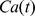, is given as:
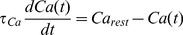
(5)and the change in release ready sites, 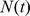, is given as:
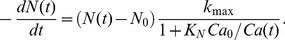
(6)During a spike, the change in Calcium concentration is:
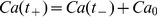
(7)and the change in release ready sites is:
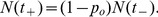
(8)Here, 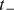 represents the time just before a spike and 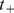 represents the time just after a spike. 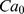 is the increase in calcium concentration after each spike. 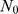 is the total number of ready sites. Initially, all the sites are release ready, 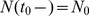, and after each spike, a fraction 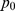 of the release ready sites are used. The synaptic strength associated with a spike at time t is proportional to 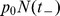, so 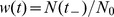.

**Figure 1:**
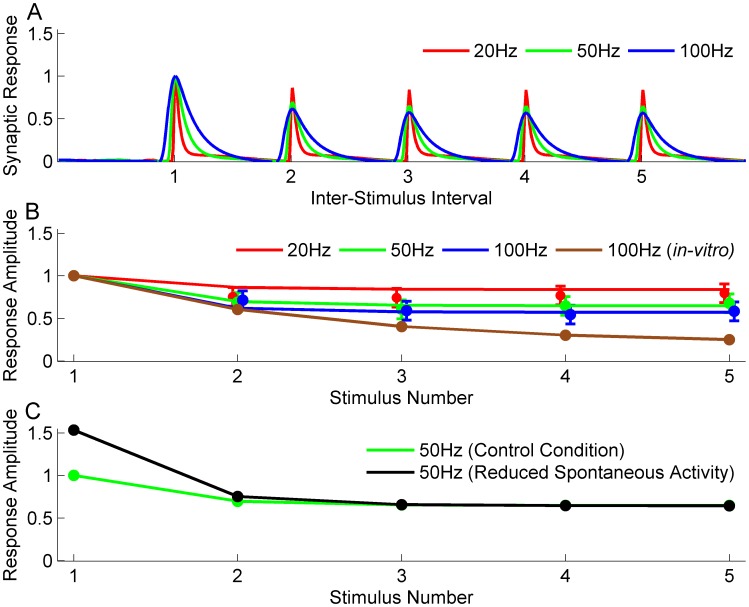
Results from our model of synaptic depression at visual thalamocortical synapses *in-vivo*, based on the model of Dittman and Regehr (1998). A) Model behavior: Dynamics of the normalized PSP’s evoked in response to stimulation of a model synapse with different frequencies. Average responses are shown to delivery of 20 Hz, 50 Hz and 100 Hz trains of electrical stimuli to LGN following the background activity. The response is normalized to equal 1 at 90% of the peak value (see Materials and Methods). Note that peaks are aligned and the response to the first stimulus is identical to all three frequencies. The *x-axis* is scaled so that inter-stimulus interval is shown as equal at all frequencies. In units of time, inter-stimulus intervals are 50 msec, 20 msec and 10 msec for the red, green and blue curves. B) Comparison with experimental data: Smooth curves show model response amplitudes (90% of peak value from panel A) as a function of stimulus number at stimulation frequencies of 20 (red), 50 (green) and 100 (blue) Hz. Mean *in-vivo* response amplitudes measured by Boudreau and Ferster [34] are indicated by colored dots; error bars show the size of the data points in their figures, which they state are larger than the error bars and thus serve as an upper bound of the experimental error bars. For comparison, results obtained by using *in-vitro* depression parameters (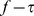 model) are shown for the case of 100 Hz stimulation (brown circles and line). C) Effect of increased intraocular pressure. Green: control response to 50 Hz stimulation, identical to green line in (B). Black: responses when background LGN firing rates were reduced before stimulation from a mean value of 11.8 Hz for control condition to a mean value of 4.1 Hz, modeling effects of increase in intraocular pressure (see Materials and Methods). The response to the first stimulus when the LGN firing rates were low is 1.5 times the value when the LGN firing rates were high. The corresponding ratio from Boudreau and Ferster [34] is 1.45±0.11.

Defining 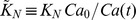, experimental data suggests that small changes in 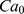 lead to measurable changes in 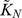 [44]. In order to allow the model to reproduce this behavior, 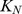 should be on the order of 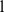. We chose 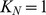. Defining 
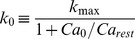
 and solving the above equations in between spikes, with *t* representing the time just after the last spike and 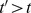 the time being solved for, the dynamics of the calcium concentration can be written as:
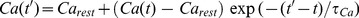
(9)while the available release ready sites can be written as:
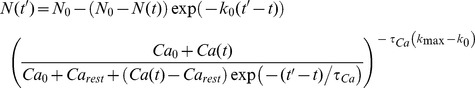
(10)
*In-vitro:* We used the 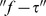 model of synaptic depression [45], [46]. Between spikes, *w* recovers toward 1 with time constant 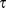: 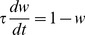. At each spike, a fraction 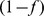 of the synaptic resources are used: 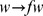. The depression parameter associated with a spike is equal to 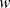 just before the spike. In agreement with Boudreau and Ferster [34], we were not able to fit the *in-vivo* data with an 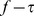 model within the error bars given in Fig. 1A. As a comparison to the model described above fit to *in-vivo* data, we used the 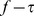 model with parameters that were fit in previous work [23] to *in-vitro* paired-pulse experiments in cat V1 slices [35]. These parameters are 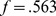, 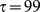 msec.

### Quantifying the PSP amplitude

To fit the *in-vivo* depression model to the data of Boudreau and Ferster [34], we equate the relative amplitude of the conductance in our model with the relative PSP amplitude. This is based on the fact that, if the synaptic conductance is small relative to the background conductance, then a scaling of the synaptic conductance vs. time by a factor just scales the potential vs. time by the same factor. As in Ref. [34], depression is characterized by PSP amplitudes relative to the first amplitude in a train. To quantify the relative PSP amplitude in our model, we follow the procedures in the experiments of Boudreau and Ferster [34]. These authors observed that the incremental amplitude of the responses to individual stimuli in each train were superimposed on slow trends. To remove the slow trends, we first interpolate (by using spline interpolation; MATLAB; Mathworks, Natick, MA) the total LGN input (as conductance) measured at each stimulus onset and subtract the fit from the original total LGN input. Then, we define the amplitude of the cortical PSP to the first stimulus as the magnitude of the conductance at 90% of the peak relative to the baseline, where baseline is defined as the average response over 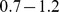 msec after stimulus onset, as in Boudreau and Ferster [34]. Subsequent PSP amplitudes are measured at the same time relative to stimulus onset as the first PSP amplitude.

### Fitting the synaptic depression model

Boudreau and Ferster [34] allowed the system to recover for periods of 1.75 sec between successive trains of stimuli. However, even with recovery between successive trains of stimuli, spontaneous activity can cause synaptic depression and affect the response of the cortical neurons to stimulation. In order to study this effect, Boudreau and Ferster used increased intraocular pressure as a means to reduce spontaneous LGN activity and studied its effect on response properties of cortical neurons. They reported that LGN spontaneous rates were 11.8±2.9 spikes/sec in the control condition and 4.1±1.4 spikes/sec in the condition of increased intraocular pressure.

We replicated these experiments in our simulations as follows: For each set of depression parameters, all of the LGN neurons in the model were made to fire spontaneously for a period of 1.75 sec. Following this, stimulus trains at 20, 50 or 100 Hz were presented (all of the LGN neurons fire synchronously, but note they have varying transmission delays as described above), and the resulting PSP’s in the cortical neuron were monitored. Each simulation leads to estimates of the train of cortical PSP amplitudes (quantified as discussed previously) at the corresponding stimulus frequency. For the control condition, we took the spontaneous firing rates of the LGN cells to be 11.8 spikes/sec. To model the effect of reduced spontaneous activity, we again allowed the LGN neurons to fire with a background rate of 11.8 spikes/sec for a period of 1.75 sec and then with a background rate of 4.1 spikes/sec for 5 sec, as in the experiments. At the end of this period, a stimulus train was presented at 50 Hz, and the resulting PSP’s in the cortical neuron were monitored. We compared the cortical PSP amplitudes from the control conditions (20, 50 and 100 Hz) and the first PSP amplitude from the reduced spontaneous activity case with the experimental data. For the reduced spontaneous activity case, Boudreau and Ferster [34] reported that the first PSP amplitude increased by 44.9
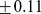
% (mean
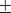
standard error) relative to control, and that subsequent PSP amplitudes were not significantly different from control. Their standard error corresponds to an error bar of 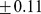 in the relative units of our Fig. 1. For the control condition, Boudreau and Ferster referred to error bars that were smaller than their displayed data points (their Fig. 4D); their data points had a radius of 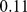 in the same relative units. We use this value of 0.11 in relative units for all error bars in our Fig. 1B and for the error bar (not shown in our Fig. 1C) for the first PSP for the reduced spontaneous activity. We searched the parameter space for 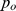 within 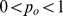, 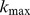 within 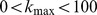, 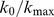 within 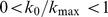, and 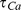 within 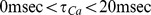, and determined parameters that agreed with the experimental data (produced values within error bars for all data points). We chose from among those the values 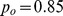, 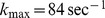, 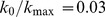 and 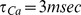, which were the parameters giving the minimum value for the maximum absolute error over the 5 stimulus trains for the spontaneous case (at 20, 50 and 100 Hz) and the first stimulus for the control case.

### Estimating the firing rate of a simple cell

To determine whether spike threshold might alter or amplify tuning effects of synaptic depression, we estimated the tuning of firing rate that would be induced by the LGN inputs. To do so, we make the simplifying assumption that voltage is linear in the excitatory conductance. For large voltage excursions, voltage response can be sublinear in the excitatory conductance [47], but the assumption of linearity can be justified for two reasons (i) From resting potential to spike threshold, i.e ∼20 mV, the relationship can be reasonably approximated as linear [47]. (ii) To the degree that voltage response is sublinear in the excitatory conductance, the problem with tuning will get worse, i.e. the difference between null and preferred responses will be weaker, so linearity is a conservative assumption for purposes of our study, which focuses on whether synaptic depression can enhance the difference between null and preferred responses.

Given the assumption that voltage response is linear in the conductance, we estimate the normalized firing rate of simple cells from the LGN input using the results of Finn et al [17]. These authors found that the firing rate of simple cells is related to mean membrane potential and its standard deviation across trials through the following power law relationship.
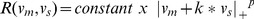
(11)Here, 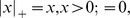 otherwise. 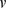 is the membrane potential relative to the resting potential, 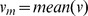, 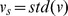, 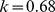 and p was found by fitting Fig. 7B of [17] to Eq. 11, giving the exponent 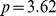.

**Figure 2:**
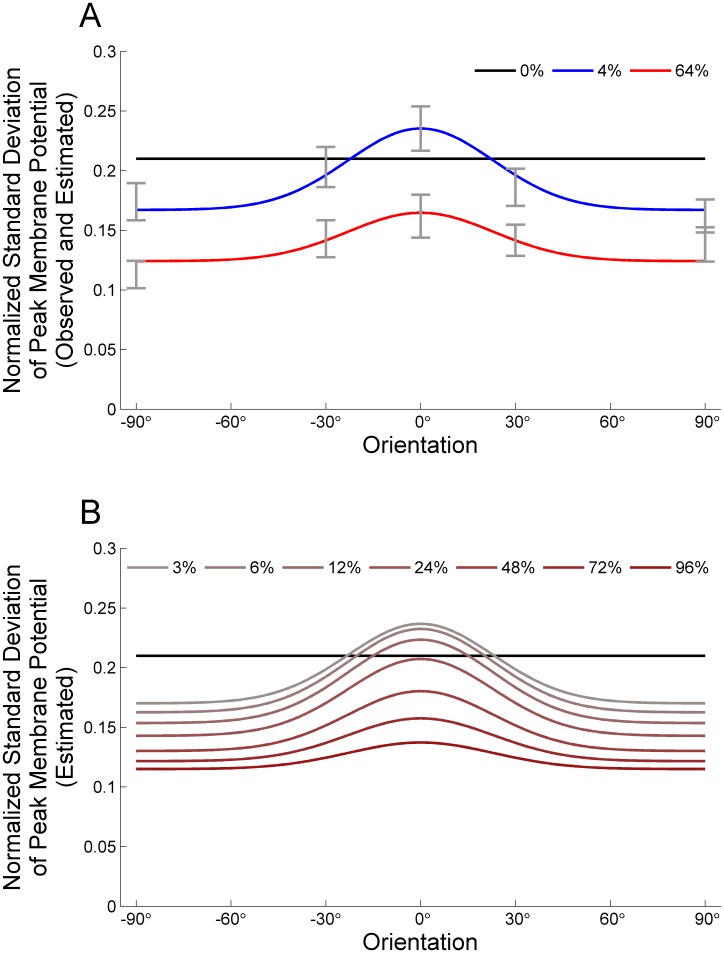
Tuning of the standard deviation of the peak membrane potential normalized to the mean peak membrane potential at 64% contrast preferred orientation. A) Data from Finn et al. [17] (data points and error bars) and our fits to them (smooth curves). Observed normalized standard deviation for the background (0% contrast) is 0.21. For 4% and 64% contrasts observed values are shown as error bars at orientations −90°,–30°,0°,30° and 90°. Estimated normalized standard deviation values are shown as smooth curves that are fit to the data by considering a single function, 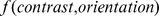, (Eq. 12), which is constrained to give observed experimental values (see Materials and Methods). 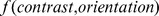 is shown here for 0% contrast (black curve), 4% contrast (blue curve) and 64% contrast (red curve). B) The estimated normalized standard deviation values, *f*(*contrast*, *orientation*), that we use in our simulations for contrasts 0%, 3%, 6%, 12%, 24%, 48%, 72% and 96%.

Supplementary Fig. 1C of [17] (replotted in our Fig. 2A, data points with error bars) shows the tuning of the normalized standard deviation of the peak voltages for three contrasts (0%, 4% and 64%) and five orientations (−90°, −30°, 0°, 30°, 90°), averaged over 52 simple cells in cat V1. For each cell, the preferred orientation was set to 0°, and the tuning curve was normalized by the cell’s peak voltage at 64% contrast at the preferred orientation. To estimate the standard deviation values for other values of contrast, *c*, and orientation, *o*, we first fit the data (Supplementary Fig. 1C of [17]) to a smooth function that we found could well fit the data, given by.
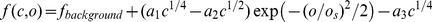
(12)Here, 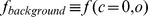 corresponds to the value at zero contrast, 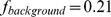 [17]. We fit the remaining constants: 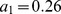, 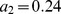, 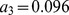 and 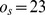
°. This function is shown as the smooth curves in Fig. 2A which are fits to the data of Supplementary Fig. 1C of [17]. The curves fall within the experimentally reported range. Next, we estimated the standard deviations at contrasts (3%, 6%, 12%, 24%, 48%, 72% and 96%; curves for these contrasts shown in Fig. 2B) and orientations (−90° to 90° with 10° intervals) to use in our simulations as follows: For each contrast *c* and orientation *o*, we generated many trials of simple cell responses and determined the mean, 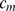, and standard deviation, 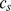, of the conductance response at each time across the cycle. We then took 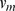 at each time equal to the corresponding value of *c_m_*. We took the values of 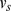 across the cycle to be the corresponding values of *c_s_* scaled so that, at the peak of the cycle, the value of 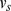, relative to the peak 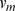 at 64% contrast and preferred orientation, was given by 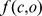 (Eq. 12). That is, we used Eq. 12 to determine 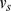 at the peak of the cycle, relative to the peak 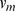 at 64% contrast and preferred orientation as determined by our simulations; and used our simulations to determine the relative values of 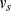 at other points in the cycle (relative to the 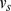 at the peak of the cycle). Then, using the 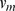 and 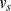 values at each point in the cycle, we used Eq. 11 to determine the normalized firing rates at each time across the cycle (normalized to the peak response across orientations and contrasts).

It is important to note that we are interested only in tuning properties and so we model normalized firing rates, ignoring absolute response levels. Given that (1) intracortical excitation does not alter mean voltage orientation tuning [6], [7], [29], [30] and (2) our model of the voltage standard deviation implicitly includes the effects of intracortical excitation (because the voltage standard deviation at the cycle peak, relative to the peak mean voltage at 64% contrast, preferred orientation, is fit to empirical measurements of voltage noise in simple cells *in-vivo*), explicitly incorporating intracortical excitation into our model would not alter spiking orientation tuning. Hence, we neglect modeling intracortical excitation in determining the spike-rate tuning that would be induced by the LGN input. Note also that, if all conductances were multiplied by a factor *k*, then using the above procedure all spike rates would be multiplied by the factor *k^p^*. Thus, only the relative conductances across contrasts and orientations, and not the absolute conductance amplitudes, influence the normalized spiking tuning that we present here.

### Quantifying orientation tuning width

We quantify orientation tuning as the half-width at half-height [17], where half-height is defined as half the distance between the maximum response and baseline (as shown in Supplementary Fig. 2 of [17]). Baseline is meant to indicate firing rate in the absence of a stimulus. We defined the baseline as the cortical-cell response when LGN-cell firing rates were set to their mean firing rates at 3% contrast, i.e. the weakest stimulus for which we had data. The rationale for this choice is that such weak stimuli typically modulate the firing rate of LGN cells linearly, without rectification. Consequently, the firing rate of LGN cells in the absence of a stimulus is approximately identical to their mean firing rate in response to a drifting grating with a 3% contrast. If the cortical cell’s response to a stimulus orthogonal to the preferred was greater than the half-height, we set the half-width at half-height to be 90^o^.

## Results

In order to assess depression of thalamocortical synapses *in-vivo*, Boudreau and Ferster [34] recorded intracellularly from V1 simple cells receiving direct input from the LGN, as judged by the short latency and low timing jitter of LGN-evoked PSP’s. They delivered trains of electrical stimuli to the LGN, and examined the change over time in the resulting PSP amplitudes recorded in the simple cell. They observed that the second PSP amplitude (*i.e.*, in response to the second stimulus in the train) was strongly reduced relative to the first PSP amplitude, with relatively little further reduction for subsequent PSP’s (see Fig. 1B). They tried to fit their data with a simple 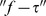 model, in which a synapse’s strength is multiplied by 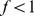 after each spike and, between spikes, relaxes toward that synapse’s maximal strength with time constant 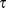. Such models have been successfully used to model cortical synaptic depression as observed in many *in-vitro* experiments [23], [45], [46]. Boudreau and Ferster [34] observed that, because of the transient character of the synaptic depression observed *in-vivo*, this model underestimated the initial depression and overestimated the depression that occurred later in the train and so gave very poor fits.

In a different set of experiments, studying the climbing fiber synapse in cerebellum, Dittman and Regehr [44] showed that recovery from depression was accelerated by accumulation of presynaptic residual calcium. They suggested a modification to the 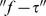 model in which the recovery time, 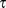, is not constant but instead is calcium dependent. This dependence leads to maintenance of synaptic efficacy under conditions that would otherwise deplete the available transmitter pool.

We find that the [44] depression model allows good fits to the *in-vivo* experimental data on monosynaptic cells obtained by Boudreau and Ferster [34] (Fig. 1). From the time course of model response to presynaptic trains of stimuli at 20 Hz, 50 Hz and 100 Hz (Fig. 1A), we can extract the amplitude of response to each stimulus. The model amplitudes (Fig. 1B, 20 Hz (red), 50 Hz (green) and 100 Hz (blue)) show reasonable matches to the experimental observations and fall within an upper bound estimate of the experimental error (Fig. 1B, error bars) for all stimulus frequencies. The model successfully captures the large initial reduction in amplitude, evident on the 2^nd^ stimulus of the train, and the small changes in amplitude over subsequent stimuli. For comparison, using parameters obtained from the 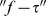 model fit from *in-vitro* experiments, we observe a much more gradual decrease in amplitude with stimulus number (Fig. 1B, 100 Hz (brown)), as noted by [34], and we found that this was true of this model for any parameters that produced significant depression.

Hereafter, we will refer to the [44] model, which uses the depression parameters from the fit to the *in-vivo* geniculocortical data of [34], as the “*in-vivo*” model, and to the 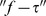 model, which uses the depression parameters from the fit of [23] to *in-vitro* geniculocortical data [35], as the “*in-vitro*” model.

The stimulus-induced depression observed by [34] was weak relative to that typically observed *in-vitro*. They speculated that this may be due to synapses already being partially depressed due to spontaneous activity, as had been suggested in the somatosensory thalamocortical system [48], [49]. To test this, they reduced LGN spontaneous firing rates by about 65% by applying intraocular pressure. Consistent with pre-existing depression, they found that this increased the amplitude of response to the first stimulus in a 50 Hz train by 45±11% on average, but had little effect on responses to subsequent stimuli in the train. We replicated this experiment by lowering the LGN firing rates by the same amounts as in the experiments, as discussed in the Materials and Methods section. The results for the *in-vivo* model closely parallel the experimental observations (Fig. 1C). Reduced spontaneous activity (Fig. 1C, 50 Hz (black)) results in increased amplitude of response to the first stimulus and has little effect on the remaining stimuli compared to spontaneous activity obtained from the control condition (Fig. 1C, 50 Hz (green)). In Fig. 1 we assume that the simple cell receives LGN inputs with weights stochastically sampled from a Gabor function (see Materials and Methods), [20], but the fit does not depend on this choice: we obtain similar results if constant weights are assigned to all LGN ON and OFF-center inputs (not shown).

Having a successful model of the depression observed *in-vivo* in cat V1, we now use this to model the effects of depression on the total LGN input received by a layer 4 simple cell at various orientations and contrasts. We assume that the simple cell receives LGN inputs with weights stochastically sampled from a Gabor function [20]. We assume that all LGN inputs have the same periodic timecourse of response to a drifting sinusoidal luminance grating of a given contrast, except that the LGN inputs vary in the phase of their response according to their positions relative to the grating and the orientation of the grating. As a model of the timecourse of LGN response, we use the responses of a single measured LGN cell to gratings of various orientations and contrasts. We study the model for eight different choices of measured LGN cell and thus eight different models of the timecourse of LGN response. Alternatively, we could have let each LGN input be modeled by a different LGN cell, but this would have tended to average out the variations; we choose the more extreme alternative in order to see the largest range of possible variations in LGN input. To study the effect of depression at thalamocortical synapses, we simply model the net LGN input to a simple cell, and do not model any cortical mechanisms, such as inhibition, that might combine with this input to produce cortical responses. To characterize the tuning of the net LGN input, we use two measures, as described in Materials and Methods. First, we characterize the tuning of the total LGN-induced conductance as a direct measure of the net input. Second, to determine whether spike threshold effects might reveal stronger effects of synaptic depression on tuning, we estimate the tuning of the simple-cell firing rate responses that would be induced by this LGN input. Note that we are only interested in tuning, which is independent of intracortical excitation [6], [7], [29], [30], and not the response amplitude, which depends on intracortical excitation [6], [7], [29], [30], so we ignore amplitude and report only normalized responses (normalized to the response at optimal orientation and largest contrast studied).

**Figure 3:**
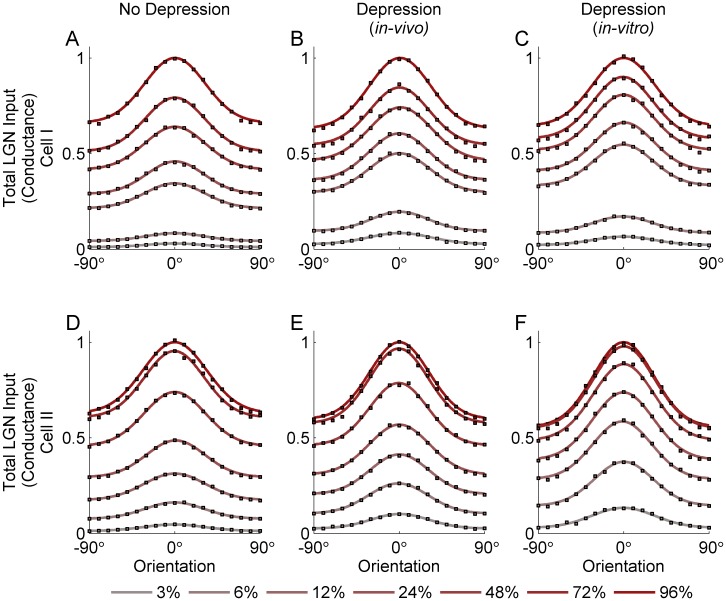
Orientation tuning of the peak LGN input to the simple cell (in terms of conductance) over a cycle in response to drifting grating stimuli of varying contrasts. Results are shown for two different models of LGN response time course, each based on the response time course of a measured LGN cell: Cell I (A–C) and Cell II (D–F). In each figure, the colored circles show peak input for 6 different contrast levels (3%, 6%, 12%, 24%, 48%, 72% and 96%) and 19 orientation angles (from −90° to 90° with steps of 10°). The colored smooth curves show fits of a Gaussian plus baseline to these tuning curves at the different contrasts. Each fit is normalized to the value of the fit at the preferred orientation at highest contrast (96%), which is set to 1. Orientation tuning curves are calculated for the cases: No Depression (A, D), Depression using *in-vivo* (B, E) fit parameters, Depression using *in-vitro* (C, F) fit parameters.

**Figure 4:**
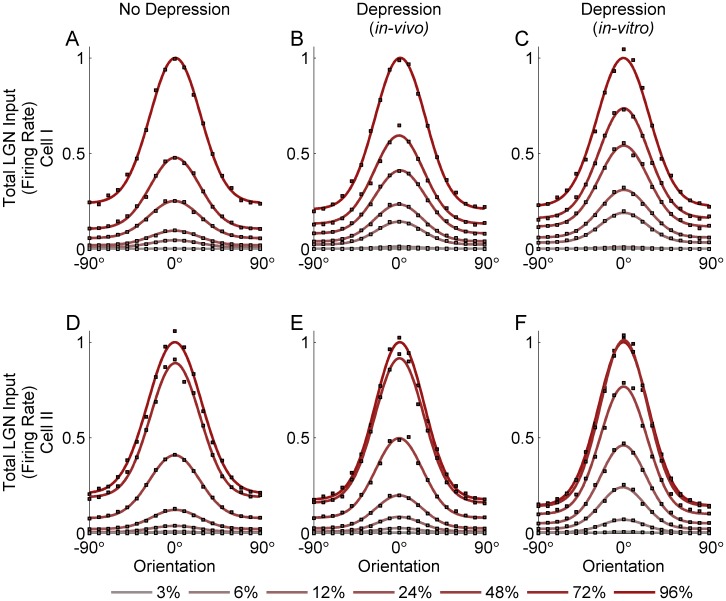
Orientation tuning of the peak LGN input to the simple cell (in terms of firing rate) over a cycle in response to drifting grating stimuli of varying contrasts. Results are shown for two different models of LGN response timecourse, each based on the response timecourse of a measured LGN cell: Cell I (A–C) and Cell II (D–F). In each figure, the colored circles show peak input for 6 different contrast levels (3%, 6%, 12%, 24%, 48%, 72% and 96%) and 19 orientation angles (from −90° to 90° with steps of 10°). The colored smooth curves show Gaussian fits to these tuning curves at the different contrasts. Each fit is normalized to the value of the fit at the preferred orientation at highest contrast (96%), which is set to 1. Orientation tuning curves are calculated for the cases: No Depression (A, D), Depression using *in-vivo* (B, E) fit parameters, Depression using *in-vitro* (C, F) fit parameters.

**Figure 5:**
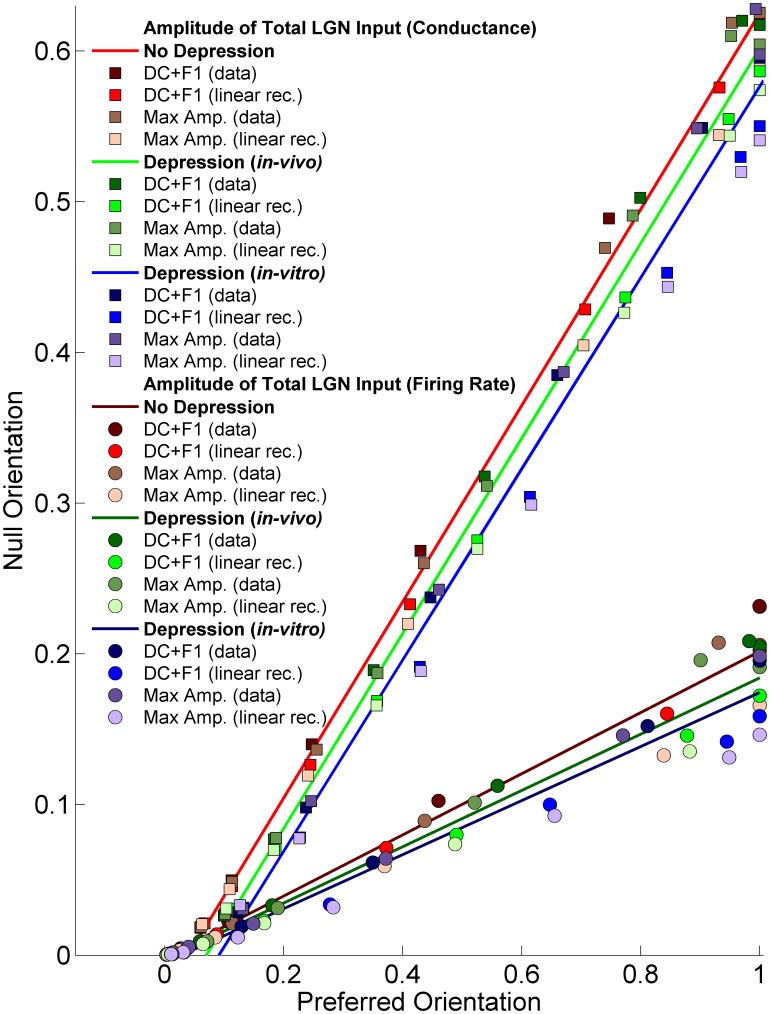
Amplitude of the conductance and the firing rate at the null orientation as a function of amplitude at the preferred orientation. The cases shown are: No Depression (red), Depression using *in-vivo* (green) or *in-vitro* (blue) fit parameters. Amplitudes are means across the 7 models of LGN timecourse, where for each model of LGN timecourse and depression the amplitude is scaled so that input to the preferred orientation at highest contrast is 1. Four different response measures are shown: either the actual experimental PSTH is used (“data”) or a linear rectified approximation to it (see Materials and Methods); and there are two different measures of the size of this response (maximum amplitude over a cycle or DC+F1, where DC is mean over a cycle and F1 is Fourier amplitude of the first harmonic). Results from all contrast levels are pooled together and displayed for each of model of depression and response measure. Linear fits (minimizing sum-squared error) to each model are also shown (See color insets).

Figs. 3 and 4 show the tuning of the peak LGN input conductance to a simple cell (Fig. 3) and of the firing rate this conductance would evoke in the simple cell (Fig. 4), for two models of LGN timecourse, each based on the response of a different measured LGN cell (cell 1: Fig. 3A-C and Fig. 4A–C; cell 2: Fig. 3D-F and Fig. 4D–F). For the conductance (Fig. 3), the input in response to the null orientation (defined as the orientation orthogonal to the preferred) grows strongly with contrast, so that the null input at high contrast is much larger than the input to the preferred orientation at low contrasts. This is true whether the synapses are modeled without depression (Fig. 3A,D) or with depression modeled as in the *in-vivo* model (Fig. 3 B,E) or the *in-vitro* model (Fig. 3 C,F). With depression, low-contrast input is larger relative to high-contrast input than without depression. This corresponds to the fact that synaptic depression induces saturation of input at lower contrasts. That is, with depression, a given low contrast is a larger percentage of the saturating contrast and hence behaves, relative to the largest contrast, as does a higher contrast without depression (note that each panel in Fig. 3 is normalized so that 1 represents the preferred input to high contrast). However, for a curve with a given size of preferred input, the size of the null input is about the same with or without depression, though slightly reduced by depression. Results for firing rate tuning curves (Fig. 4) are similar in these respects, although the tuning curves for the firing rate are narrower and the null firing rate much lower relative to preferred firing rate than was the case for membrane potential (Fig. 3). Overall, as shown in Fig. 5, the ratio of null input to preferred input changes little across contrasts, both in terms of membrane potential and firing rate, and synaptic depression of either form causes only a very slight reduction in this ratio. Depression fit to the *in-vitro* data causes about twice the reduction in this ratio as does depression fit to the *in-vivo* model, but both reductions are slight.

**Figure 6:**
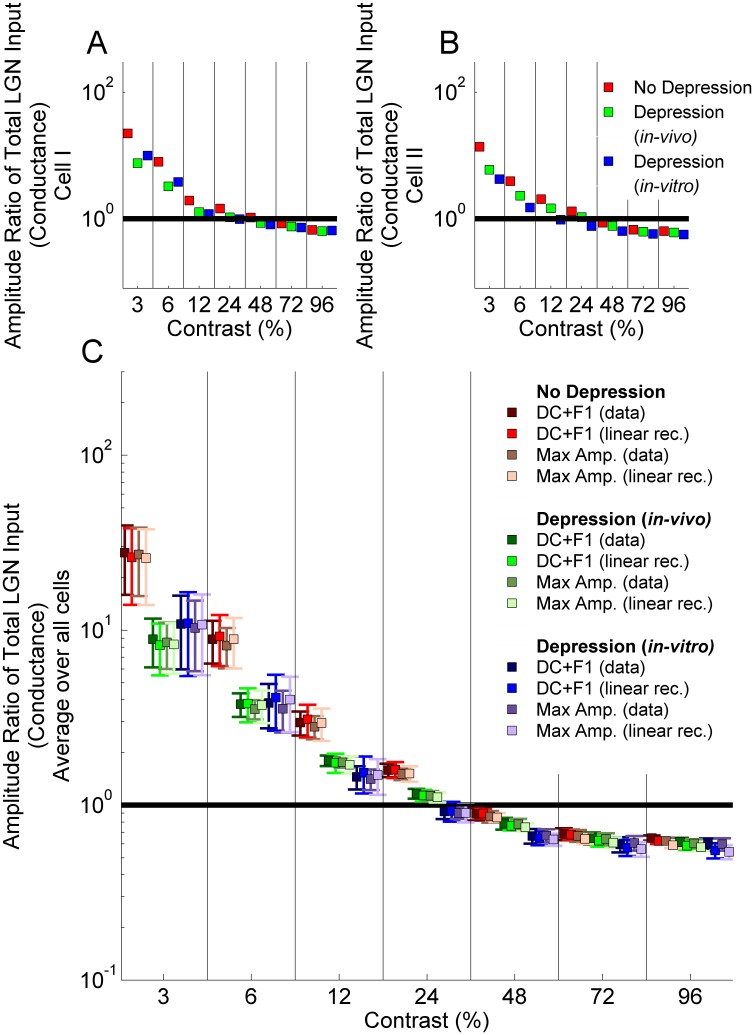
Ratio of the amplitude of the tuning curve in Fig. 3 (normalized conductance) at the null orientation (the orientation orthogonal to the preferred) at 96% contrast to that at the preferred orientation (*x-axis*; 3%, 6%, 12%, 24%, 48%, 72% and 96%). Black horizontal line indicates amplitude ratio 1. The cases shown are: No Depression (red), Depression using *in-vivo* (green) fit parameters, Depression using *in-vitro* (blue) fit parameters. A) Amplitude ratios for Cell I of Fig. 2; B) Amplitude ratios for Cell II of Fig. 2; C) Amplitude ratios averaged over all 7 experimentally measured LGN cells (mean ± std error). In C, four different response measures are shown: either the actual experimental PSTH is used (“data”) or a linear rectified approximation to it (see Materials and Methods); and there are two different measures of the size of this response (maximum amplitude over a cycle or DC+F1, where DC is mean over a cycle and F1 is Fourier amplitude of the first harmonic); see inset for colors corresponding to these 4 measures. All points between each pair of vertical bars represent the same contrast: for different response measures, results for each contrast are offset relative to each other for ease of visualization.

**Figure 7:**
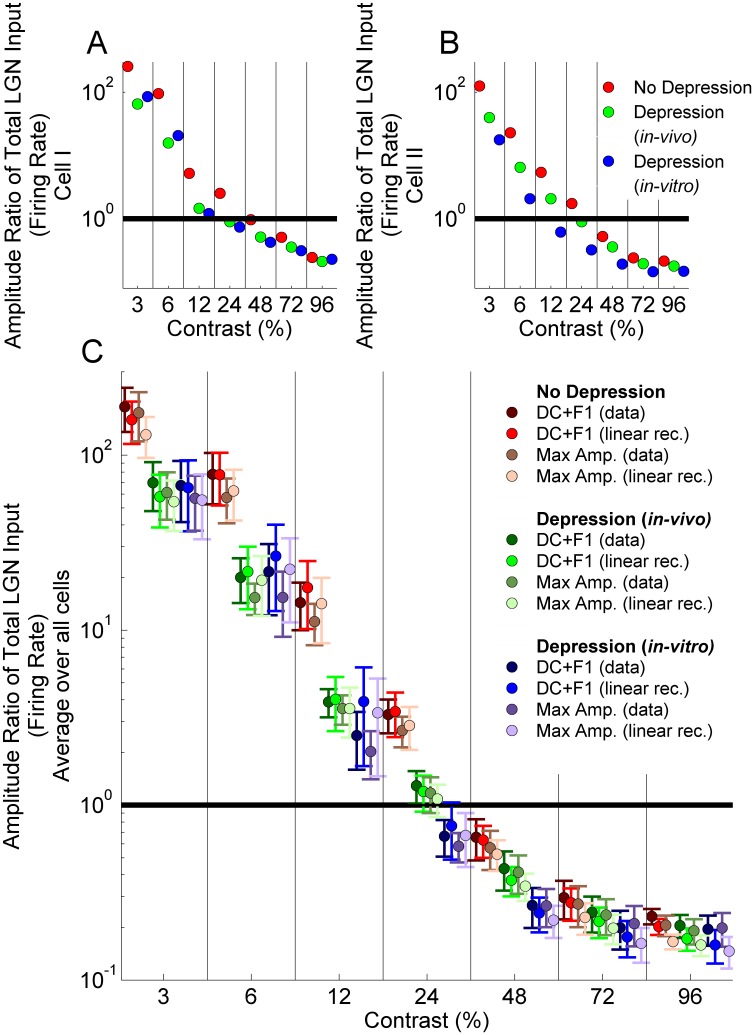
Ratio of the amplitude of the orientation tuning curve in Fig. 4 (normalized firing rate) at the null orientation (the orientation orthogonal to the preferred) at 96% contrast to that at the preferred orientation at varying contrasts (*x-axis*; 3%, 6%, 12%, 24%, 48%, 72% and 96%). Black horizontal line indicates amplitude ratio 1. The cases shown are: No Depression (red), Depression using *in-vivo* (green) fit parameters, Depression using *in-vitro* (blue) fit parameters. A) Amplitude ratios for Cell I of Fig. 2; B) Amplitude ratios for Cell II of Fig. 2; C) Amplitude ratios averaged over all 7 experimentally measured LGN cells (mean ± std error). In C, four different response measures are shown: either the actual experimental PSTH is used (“data”) or a linear rectified approximation to it (see Materials and Methods); and there are two different measures of the size of this response (maximum amplitude over a cycle or DC+F1, where DC is mean over a cycle and F1 is Fourier amplitude of the first harmonic); see inset for colors corresponding to these 4 measures. All points between each pair of vertical bars represent the same contrast: for different response measures, results for each contrast are offset relative to each other for ease of visualization.

To better assay the effect of the null-orientation response on orientation tuning, we examine the ratio of the response to the null orientation at the highest contrast to that at the preferred orientation at a given contrast (Fig. 6, membrane potential; Fig. 7, firing rate; contrast of preferred-orientation stimulus is given on the *x-axis*; different measures of response in figures discussed further below). As noted in the Introduction, in real neurons this ratio generally stays below 1 even for the lowest-contrast preferred-orientation stimulus. It must stay below 1 to explain the observation that cells show spike responses to the preferred orientation at the lowest responsive contrast but not to the null orientation even at the highest contrast. For the two LGN cells examined in Figs. 3,4 (Figs. 6A,B and Figs. 7A,B) and more generally on average across the eight LGN cells used as models of LGN time course (Figs. 6C, 7C), the results using *in-vivo* vs. *in-vitro* depression models are very close (see error bars in Figs. 6C, 7C). Depression improves the results (reduces the ratio) at any given preferred-orientation contrast, but ratios remain greater than 1 for low preferred-orientation contrasts under all depression models (for contrasts of 12% or less for the *in-vitro* depression model, and contrasts of 24% or less for the *in-vivo* depression model or for no depression). Although the contrast levels at which the ratio is less than 1 for firing rate responses (Fig. 7) are similar to those for conductance responses (Fig. 6), the range of the ratio for different contrasts is much broader for firing rate responses.

**Figure 8:**
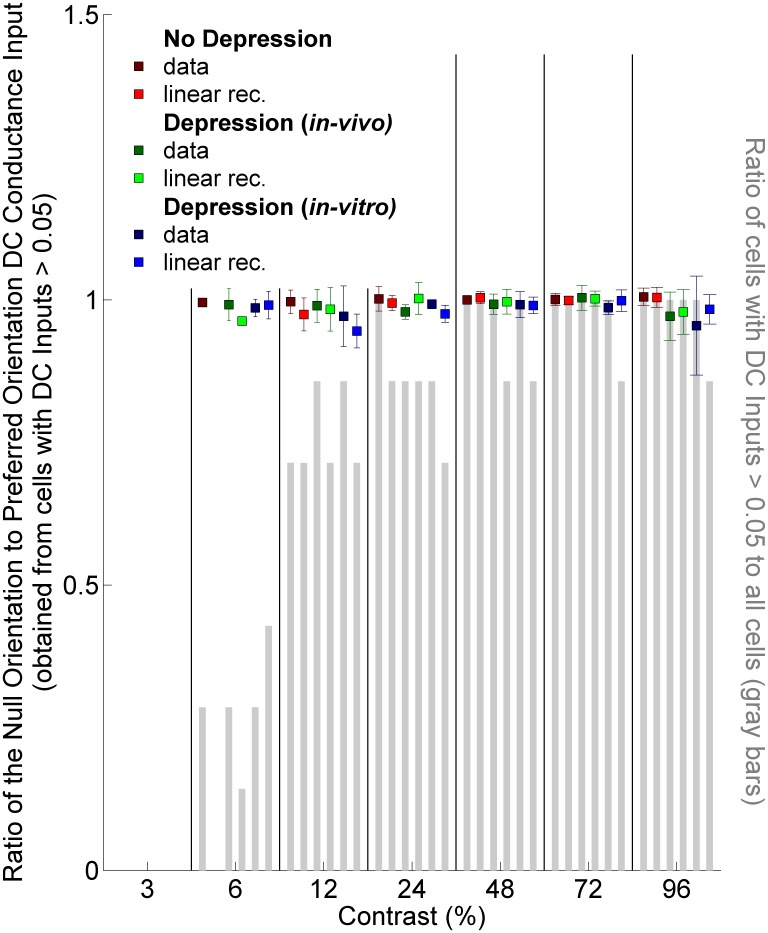
Ratio of the null orientation DC input to preferred orientation DC input averaged over LGN cells, across contrasts. Only cells for which both DC’s were greater than 0.05 are included in the calculation; mean ratios (squares) and the standard deviations (error bars) are shown. The cases shown are: No Depression (red), Depression using *in-vivo* (green) or *in-vitro* (blue) fit parameters. For each case, two different response measures are shown: either the actual experimental PSTH is used (“data”) or a linear rectified approximation to it (see Fig. 7 legend and Materials and Methods). For a given response measure and contrast (3%, 6%, 12%, 24%, 48%, 72% or 96%), ratio of number of cells that have both DC inputs >0.05 to total number of cells is shown as gray bars.

As discussed in the Introduction, the relative suppression of response to null-orientation stimuli, which is critical to observed orientation tuning, also manifests as a value significantly less than 1 for the ratio of the mean LGN-induced conductance to a null-orientation stimulus to that for a preferred-orientation stimulus, at least at high contrast. We examined this for our various models. We found that the ratio remains very close to 1 across all contrasts and regardless of the absence or, if present, the form of geniculocortical depression (Fig. 8). In this figure, at each contrast we averaged only over LGN cells for which the DC component of the null-orientation response and the DC component of the preferred-orientation response were both >0.05, in units in which the peak conductance to the highest-contrast preferred orientation is 1 (as in Fig. 3). When these mean responses were smaller, the ratios of the two responses could be poorly behaved, but the absolute difference between the two mean responses was never greater than 0.02 across the excluded cases.

**Figure 9:**
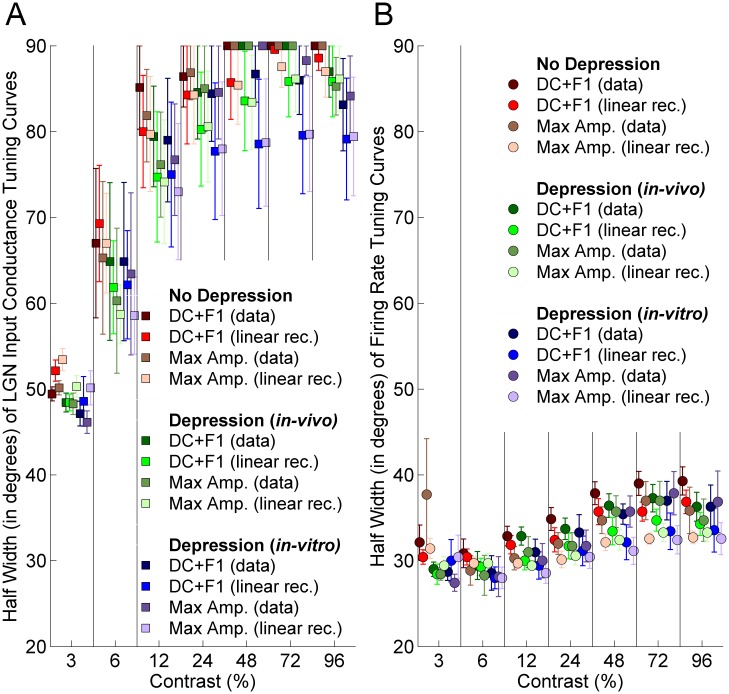
Half-width of tuning curves at half-height (i.e., difference between orientation giving peak response and orientation giving 50% of peak response) obtained from the tuning curves of total LGN input: A) Conductance B) Firing Rate. Each half-width is calculated at a specific contrast (*x-axis*; 3%, 6%, 12%, 24%, 48%, 72% and 96%). Half-widths are averaged over the experimentally measured LGN cells, mean ± std error (*y-axis*). If null-orientation response was greater than 50% of the peak response, the half-width is undetermined (half-width >90°, See Supplementary Fig. 2 of [17); in such cases we set the half-width to 90°. Four different response measures are shown, as described in legend of Fig. 7. Conventions as in Fig. 7.

Finally, we examined the dependence on contrast of the half-width at half-height (Materials and Methods) of the tuning curve (Fig. 9). Experimentally, half-width is invariant to stimulus contrast for firing rate responses [17], [32], [33], [50], while for voltage responses it broadens moderately with contrast (this broadening, along with the changing input/output relationship due to decreasing voltage variance, produces the contrast-invariant spiking response) [17], [19], [50]. For conductance responses, the half-width increases dramatically with contrast, reaching a plateau near 90° for contrasts of 24% and above, reflecting the lack of suppression of the untuned component of the LGN input (Fig. 9A). This increase is slightly less with synaptic depression, with a greater reduction for *in-vitro* than for *in-vivo* depression, but the reductions due to depression are slight. As a result, firing-rate tuning widths increase significantly with contrast (Fig. 9B). The tuning widths are slightly lower with synaptic depression, but the increase in width with contrast is similar with or without depression.

Previous modeling efforts have used a linear rectified model of LGN input and have used the DC+F1 as a surrogate for the peak input [24]. To determine whether these approximations affect any of our results, in Figs. 5–9, we computed results both with and without these approximations. To model the response of an LGN cell, we considered the cycle-averaged PSTH (referred to as “data” in Fig. 6C, 7C), or a linear-rectified fit to this PSTH consisting of a sinusoidal modulation plus a constant, with negative values set to zero (see Materials and Methods). For each of these two models, we considered two measures of the total input to the simple cell: the peak input over a cycle, or the sum of the mean (DC) and first harmonic (F1) over a cycle, which would be equal to the peak if there were no higher harmonics. As can be seen in the figures, these alternative choices for measures of LGN response or of the summed LGN input make very little difference to the results.

## Discussion

We find that synaptic depression as measured *in-vivo* in thalamocortical synapses [34] can be well fit by a model of depression that incorporates a calcium-dependent change in the time constant of recovery from depression [44]. Using this “*in-vivo*” model as well as an earlier, simpler model [45], [46] fit to *in-vitro* data [35], we find that thalamocortical depression in either form has little impact on the orientation tuning of the LGN input to V1 simple cells or its contrast dependence, other than to induce response saturation at lower contrasts. Thus, the contrast-invariance of the orientation tuning of simple cell responses [17], [32], [33], [50] must depend on cortical mechanisms beyond the LGN synapse, e.g. feedforward inhibition driven by LGN excitation of cortical inhibitory cells [20], [25], [26], [51] and/or effects of spike threshold and its interaction with voltage fluctuations [17], [26], [50].

### Modeling synaptic depression

In our fit to the *in-vivo* data with the calcium-dependent recovery model, we found the exponential decay time constant for calcium to be 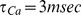, which is very low in comparison with/found by Dittman and Regehr [44], at the climbing fiber to purkinje cell synapse (or 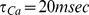 when EGTA-AM was added to the presynaptic terminals). In addition to the difference in the synapses being studied, one possible contributor to the difference is temperature: Dittman and Regehr’s experiments were conducted at 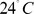
_,_ whereas Boudreau and Ferster’s were at 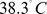. A more accurate model of synaptic depression may also include the dependence of temperature as observed experimentally [52]. It also may be that the model, including the time constant necessary to fit the thalamocortical data, does not accurately represent the underlying biophysical mechanisms at the thalamocortical synapse, but is simply a phenomenological model that is able to fit the data.

### The Role of Synaptic Depression in V1 Responses

We have found that synaptic depression causes little change in the ratio of null-orientation to preferred-orientation LGN input to a simple cell (Fig. 5) and thus has little effect on orientation tuning, other than to cause saturation to occur at lower contrasts as reported previously [23], [27].

It is not surprising that synaptic depression causes similar suppression of response to all orientations at a given contrast, and thus has little effect on orientation tuning. Individual LGN cells are not tuned for the orientation of a drifting grating, and thus depression has the same effect on an individual LGN cell’s output for any stimulus orientation. The LGN response is confined to and peaks within a particular portion of the stimulus cycle. Synaptic depression changes the temporal waveform over a cycle of the input from an LGN cell, relative to its firing rate, by suppressing later portions of the response relative to earlier portions (e.g., [23] (Fig. 5)). Orientation tuning arises from the fact that a preferred-orientation stimulus tends to drive all of a simple cell’s LGN inputs to peak near the same phase of the stimulus cycle, whereas in response to a null grating LGN responses are dispersed across the stimulus cycle [20]. The total LGN input to a simple cell over time is predicted to be well approximated, as we have verified here, by the rectified sum of a mean (the zeroeth harmonic) and a sinusoid (the first harmonic or F1; the temporal modulation of the input at the same temporal frequency as the stimulus), in essence because the Gabor-function receptive field filters out higher harmonics in the time course of individual LGN cells [24]. The mean is untuned for orientation, and represents the sum of the means of the individual LGN inputs. The F1 is the orientation-tuned component, and represents the tendency of the LGN cells to all modulate their firing rates at roughly the same phase for preferred but not for null stimuli. For synaptic depression to differentially affect response to different orientations, the change in the LGN input waveform that it induces must either change the size of the DC relative to the F1 (*e.g.,* a thinner pulse of input has a smaller individual DC/F1 ratio) and/or change the tuning of the F1 (*i.e.,* lead to greater decrease in the net F1 for a given degree of phase desynchronization). Both effects are apparently small, even for strong depression.

Previous papers have studied the effects of synaptic depression on orientation selectivity. Carandini et al. [27] assumed perfect push-pull inhibition, i.e. that for each LGN cell driving excitatory input to a simple cell, an identical LGN cell except of opposite center type drives equal-strength inhibitory input. This eliminates the untuned component of the LGN input, leaving only the tuned component, so that in particular the input to a null-orientation stimulus is zero. Given a nearly-zero spike threshold, this solves the problem of achieving contrast-invariant orientation tuning [20], leaving synaptic depression simply to solve the problem of causing earlier contrast saturation in cortex than in LGN without altering orientation tuning.

Our results and conclusions significantly differ from Banitt et al. [28], who also studied the effect of synaptic depression on orientation tuning. First, we focused on the effects of thalamocortical synaptic depression as measured *in-vivo* by Boudreau and Ferster [34]. We fit our model to the full set of *in-vivo* data (including 20, 50, and 100 Hz pulse train data taking into account prior spontaneous activity, as well as the case of reduced spontaneous activity). The “moderate depression” model of Banitt et al. [28] was said to be fit to this *in-vivo* data, and in agreement with our findings it failed to suppress non-preferred responses. However their moderate depression model, while reasonably fitting the *in-vivo* data for 50 Hz pulse trains, failed to fit the data for 20 Hz pulse trains (their Fig. 6; conversely, their “strong depression” model in that figure reasonably fit the first two ISI’s for 20 Hz *in-vivo* data, but failed to fit the 50 Hz *in-vivo* data); they did not illustrate model fits to 100 Hz *in-vivo* data. It also appears that their fitting to the *in-vivo* data was done assuming an undepressed state as the initial condition, rather than a partially depressed state due to prior spontaneous activity as in the data, as no mention of spontaneous activity was made for this protocol.

Second, Banitt et al. [28] primarily focused on a “strong depression” model that was said to be based on a fit to the *in-vitro* depression data of Stratford et al [35] (Fig. 1g). They reported that this strong depression could largely suppress depolarization at orientations far from the preferred and achieve contrast-invariant spiking tuning, unlike our findings using a depression model fit to the same *in-vitro* data. However, they appear to have used a different strong depression model to study orientation tuning than was used to fit the *in-vitro* data, and both appear to differ from the strong depression model as defined parametrically, even though all are identified as the same strong depression model. After a 20 msec ISI beginning from an undepressed state, their model fit to the *in-vitro* data matched the observed depression to 62% of initial strength (their Fig. 3C); their model used in studying orientation tuning depressed to 44% of initial strength (their Fig. 6B); and it is easy to calculate that the parameters they gave for the strong depression model would produce synaptic depression to about 30% of initial strength, which should manifest as a similar level of EPSP depression (because their model was linear except for somatic voltage-dependent conductances and reversal potential effects, none of which should have led to significant deviations from linearity for the small depolarizations of an EPSP). These discrepancies were not noted or explained. It is also puzzling that their moderate depression model (their Fig. 9A) produced more hyperpolarized voltages than their strong depression model (their Fig. 8C) for non-preferred orientations at low contrasts (3% and 10%), even though their moderate depression model appeared to always produce less synaptic depression at the thalamocortical synapses than their strong depression model (*e.g*., their Fig. 6). Without knowing the models they actually used to study orientation tuning, we find it difficult to compare their findings to ours. In sum, we conclude that depression models matched to either *in-vivo* or *in-vitro* physiological data, which we have constructed, do not significantly impact orientation tuning.

Thalamocortical synaptic depression has also been postulated to contribute to other V1 response properties, including band-pass rather than low-pass responses to sinusoidal stimuli, nonlinear differences in the responses to broadband or transient stimuli vs. the responses to sinusoidal stimuli, contrast adaptation, and direction selectivity [53]; contrast-dependent changes in temporal frequency tuning [23] and in the temporal phase of response to drifting grating stimuli [23], [53]; and the suppression of responses to preferred stimuli by superimposed mask stimuli [27]. It will be of interest to revisit these issues with a model matched to the thalamocortical depression observed *in-vivo*.

### Applicability to Rodents

We have based our model on a large number of studies in cat V1 (see references in Introduction) that have established the classic “Hubel-Wiesel” model of the arrangement of LGN inputs to layer 4 simple cells, the dependence of spiking tuning for orientation and contrast on the tunings of the voltage mean and voltage standard deviation and on feedforward vs. intracortical input, and the properties of synaptic depression *in-vivo* and *in-vitro*. While we expect our basic finding, that synaptic depression does not significantly impact orientation tuning or its contrast dependence, will carry over to rodents, many of these details may differ in rodent V1. In particular, it is already known that ON-center and OFF-center excitatory inputs appear to be more strongly overlapping in rodent [54] vs. cat [10], [14], [55] simple cells, with spatially “intervening inhibition” playing an important role in segregating ON from OFF subregions in rodents [54]; cats have a “push-pull” arrangement between excitatory and inhibitory input, whereas in rodents the arrangement appears to be “push-push” [47], [56]; and the difference between orientation tuning in LGN and V1 is much less marked in rodents than in cats [57]. Further investigation is needed to determine the degree to which the mechanisms of orientation selectivity and its contrast invariance differ between carnivores and rodents.

### Origins of Contrast Invariance of Orientation Selectivity

As summarized in the Introduction, the origin of contrast-invariant orientation tuning in thalamic-recipient simple cells in middle layers of anesthetized cat V1, in response to drifting gratings, can be attributed to: (1) the Gabor-function arrangement of LGN inputs to a simple cell [5], which causes the net LGN input to be composed of an orientation-untuned DC and an orientation-tuned F1 [20], [24]; (2) some degree of suppression of the voltage response to a null-orientation stimulus relative to that to a preferred-orientation stimulus, relative to the levels expected from LGN input alone [17], [19]; and (3) contrast-dependent suppression of voltage noise, causing the mean voltage in response to a null stimulus to stay the same number of standard deviations from threshold across contrasts and thus preventing spiking response to the null orientation [17], [19]. Sadogapan and Ferster [19] presented evidence that (3) is substantially due to a corresponding decrease in the variability of the LGN input to simple cells with increasing stimulus contrast. Several network mechanisms that could cause such variability of suppression have also been proposed [58]–[60]. The mechanism responsible for (2) is not known. One postulated mechanism for (2) is an untuned component of feedforward inhibition that grows with contrast [20], [24], [26], [27], which might also contribute to (3) by decreasing input resistance. The present work demonstrates that thalamocortical synaptic depression matched to physiological measurements does not substantially contribute to (2) or more generally to contrast-invariant orientation tuning.

## Supporting Information

Dataset S1 **LGN Firing Rates (Hz).** Each.csv file is from a single LGN cell. Each file contains a matrix. Rows: Time (Duration: 625 msec (1 cycle)). Columns: Contrast (Total 7∶96%, 72%, 48%, 24%, 12%, 6%, 3%).
(ZIP)
